# Up-regulation of expression and lack of 5' CpG island hypermethylation of *p16 INK4a *in HPV-positive cervical carcinomas

**DOI:** 10.1186/1471-2407-7-47

**Published:** 2007-03-14

**Authors:** Tatiana A Ivanova, Daria A Golovina, Larisa E Zavalishina, Galina M Volgareva, Alexey N Katargin, Yulia Y Andreeva, Georgy A Frank, Fjodor L Kisseljov, Natalia P Kisseljova

**Affiliations:** 1 Institute of Carcinogenesis, N.N. Blokhin Cancer Research Center, Russian Academy of Medical Sciences, Moscow 115478, Russia; 2 P.A. Gertzen Institute of Oncology, Russian Ministry of Health, Moscow 125284, Russia; 3 Singapore National Cancer Centre, Singapore, 169610, Republic of Singapore

## Abstract

**Background:**

High risk type human papilloma viruses (HR-HPV) induce carcinomas of the uterine cervix by expressing viral oncogenes E6 and E7. Oncogene E7 of HR-HPV disrupts the pRb/E2F interaction, which negatively regulates the S phase entry. Expression of tumor suppressor p16^ink4a ^drastically increases in majority of HR-HPV associated carcinomas due to removal of pRb repression. The p16^ink4a ^overexpression is an indicator of an aberrant expression of viral oncogenes and may serve as a marker for early diagnostic of cervical cancer. On the other hand, in 25–57% of cervical carcinomas hypermethylation of the *p16 INK4a *promoter has been demonstrated using a methylation-specific PCR, MSP. To evaluate a potential usage of the *p16 INK4a *5' CpG island hypermethylation as an indicator of tumor cell along with p16^ink4a ^overexpression, we analyzed the methylation status of *p16 INK4a *in cervical carcinomas

**Methods:**

Methylation status of *p16 INK4a *was analyzed by MSP and by bisulfite-modified DNA sequencing. The expression of p16^ink4a ^was analyzed by RT-PCR and by immunohistochemical technique.

**Results:**

The extensive methylation within *p16 INK4a *5' CpG island was not detected either in 13 primary cervical carcinomas or in 5 cancer cell lines by bisulfite-modified DNA sequencing (including those that were positive by MSP in our hands). The number and distribution of rare partially methylated CpG sites did not differ considerably in tumors and adjacent normal tissues. The levels of the *p16 INK4a *mRNA were increased in carcinomas compared to the normal tissues independently of the number of partially methylated CpGs within 5'CpG island. The transcriptional activation of *p16 INK4a *was accompanied by p16^ink4a ^cytoplasmic immunoreactivity in the majority of tumor cells and presence of a varied number of the p16 positive nuclei in different tumors.

**Conclusion:**

Hypermethylaion of the *p16INK4a *5' CpG island is not a frequent event in HR-HPV-positive cervical carcinomas and cannot be an effective marker of cancer cells with up-regulated expression of p16^ink4a^. Our data confirm other previous studies claiming specific *p16INK4a *up-regulation in the majority of cervical carcinomas at both the protein and mRNA levels. Cytoplasmic accumulation of p16^ink4a ^is a feature of cervical carcinomas.

## Background

The inhibitor of cyclin-dependent kinases p16^ink4a ^is a component of p16^ink4a^-Cdk4-6/cyclinD-pRb signaling pathway, which is perturbed in many tumors. In normal cells p16^ink4a ^functions as a negative regulator of cell cycle progression by controlling the activity of tumor suppressor pRb. P16^ink4a ^inhibits phosphorylation of pRb by cyclinD-Cdk4/Cdk6 complexes. Hypophosphorylated pRB binds to E2F transcription factors and inhibits them in G0 and early G1 phases of cell cycle. In proliferating cells phosphorylation of pRb releases E2F and induces genes that mediate S phase entry. In tumor cells the pRB-E2F interaction may be distured by different ways, including mutations or inappropriate phosphorylation of pRb due to Cdk4 orCdk6 kinases overexpression or loss of the p16^ink4a ^inhibitor [1]. The function of p16^ink4a ^may be lost due to the mutations or suppression of the transcription by promoter methylation in different type of tumors [2-4]. As a rule, simultaneous inactivation of both pRb and p16^ink4a ^does not occur in a single tumor [5,6]. A regulatory feedback exists in the pRb/p16 pathway: transcription of *p16 INK4a *is repressed by p Rb. In tumor cells lacking the pRb function the *p16 INK4a *transcription is activated due to the removal of the pRb repression. [7,8]. The p16^ink4a ^overexpression cannot inhibit cell cycle progression in these tumor cells in the absence of pRb.

Mechanism for inactivation of p16^ink4a^/pRb signaling pathway in HR-HPV-positive cervical tumors differs from that in virus-negative tumors. High risk type human papilloma viruses induce carcinomas by expressing viral oncogenes E6 and E7. Infections with HR-HPVs are extremely widespread in the world but malignant transformation is a rare consequence of infection and is associated with the aberrant expression of viral oncogenes in basal and parabasal epithelial cells [9,10]. Continued E6/E7 expression at a relatively high level is necessary for the maintenance of the transformed phenotype of epithelial cells [11,12]. Oncogene E7 of HR-HPV disrupts pRb/E2F interaction, releases active E2F and induces the pRb degradation through a proteasome-dependent mechanism [13,14]. Thus, the level of pRb is decreased drastically and the protein is not revealed by immunohistochemical technique in the majority of cancer cells in HR-HPV-positive cervical tumors [6,15]. Due to the unified mode of inactivation of the pRb/p16 pathway and existence of the regulatory feedback the vast majority of HR-HPV-positive cervical tumors display strong a p16^ink4a ^expression. The p16^ink4a ^overexpression is an indicator of aberrant expression of viral oncogenes and HPV-induced transformation of epithelial cells [10]. Several research groups (including us) have demonstrated by immunohistochemical technique a manifold increase of the p16^ink4a ^expression in cervical dysplasia and carcinomas in comparison with that in normal epithelium [16-19] There is an abundant evidence demonstrating that immunohisto-/cytochemical detection of the protein p16^ink4a ^is a promising test for revealing the HPV-infected cells with malignant potential [[10,20], for review see [21]]. Thus, the detection of the protein p16^ink4a ^overexpression is especially useful for early diagnostics of HR-HPV-associated cervical cancer.

Along with the detection of p16^ink4a ^expression in the majority of the HR-HPV-positive cervical tumors, hypermethylation of *p16 INK4a *promoter has been demonstrated in 25–57% of cervical carcinomas by methylation-specific PCR, MSP [22-27]. Hypermathylation of *p16 INK4a *promoter is a well known mechanism of suppression of the gene transcription [4,5]. The co-existence of both p16^ink4a ^overexpression and hypermethylation of gene promoter in a cervical cancer needs to be further examined.

To evaluate potential of the *p16 INK4a *5' CpG island hypermethylation usage as an indicator of tumor cell along with p16^ink4a^expression, we analyzed the methylation status of the *p16 INK4a *promoter region by MSP and bisulfite sequencing and expression by RT-PCR and immunohistochemical technique in HR-HPV-positive cervical tumors and cervical carcinoma cell lines.

## Methods

### Tissue specimens

Twenty six squamous cell carcinomas (SCC) of uterine cervix and 14 specimens of adjacent normal tissues from patients with I, II and III FIGO stages of disease were collected under the approval of the Institutional Review Board of the Blochin Cancer Research Center. Informed consent was obtained from the patients prior to surgery. All tumors were HPV16/18-positive and E7 viral genes were expressed in all of them according to PCR analysis.

### Cell lines

The human cervical carcinoma cells HeLa, SiHa, CaSki, C-4-I and C33A (American Type Culture Collection, Rockville, MD) were maintained in DMEM supplemented with 10% FCS and 2mM glutamine.

### Immunohistochemistry

Serial sections (4–5 μm thick) of neutral formalin-fixed and paraffin embedded tissues were used. The first section was stained with hematoxylin-eosin to confirm the diagnosis. Three independent pathologists (G.F., L.Z. and Y.A.) verified the diagnosis. Next sections were stained with antibodies. Normal cervical epithelium was included into every set of slides for staining as a negative control [18]. The procedures of epitope retrieval, blocking of nonspecific binding and endogenous peroxidase activity, incubation with the p16^ink4a^-specific monoclonal antibodies (CINtec™ Histology Kit, DakoCytomation) were conducted according to Klaes et al [17]. To visualize the results, the slides, which had been exposed to the primary antibodies, were treated with the EnVision™ +Dual Link DakoCytomation reagent. The reaction was developed with either AEC or DAB chromogen. The slides were counterstained with Mayer's hematoxylin. To detect p16^ink4a ^in cells, they were grown on the HistoBond adhesive coated slides (SMT Geraetehandel GmbH, Germany), fixed with 4% paraformaldehyde, washed with PBS and processed similarly to the deparaffinized histological slides.

### DNA and RNA preparations

DNAs and RNAs were isolated from frozen tissues or cells by homogenization in the 4, 5 M solution of guanidine isothiocyanate and centrifugation through cesium chloride cushion as described previously [28].

### Bisulfite based cytosine methylation analysis of *p16 INK4a*

**Figure 1:**
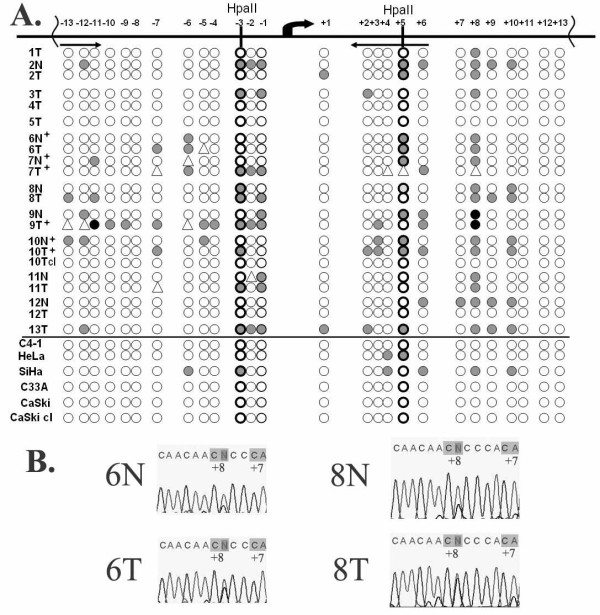
**Bisulfite-modified sequencing analysis of *p16 INK4a *5' CpG island in cervical carcinomas and cervical cancer cell lines**. A. Schematic representation of the *p16 INK4a *5' region: broken arrow indicates the location of the translation start site; figures above horizontal line indicate the numbers of CpG dinucleotides relative to the translation start site; vertical lines indicate Hpa II recognition sites; horizontal arrows indicate positions of primers for MSP analysis [30], + indicates MSP-positive samples; each row of circles represents a sequence of total PCR product generated from bisulfite-treated DNA of tumor (T) and normal adjacent tissue (N) or sequence of cloned PCR product (cl), one clone out 5 identical clones is presented; figures on the left side of scheme indicate specimen numbers; open circles – unmethylated cytosine, gray circles – partially methylated cytosine, black circles – methylated cytosine, triangles – status of cytosine is not detected. B. Chromatograms showing CpG-dinucleotides with unmethylated and partially methylated cytosines in some normal and tumor tissues. Numbers of CpG-dinucleotides and samples are indicated, as in A.

DNAs (1.5–3.0 μg) were denaturated by incubation in solution of 0.35 M NaOH for 10 minutes at 37°C. The reaction of bisulfite conversion was carried out in a solution of sodium bisulfite (3.1 M) and hydroquinone (0.6 mM) at pH 5.5 and 50°C during 4 hours. After desalting DNAs were desulfonated by incubation in solution of 0.3 M NaOH at 37°C for 10 min, then the solution was neutralized with 5 M ammonium acetate (pH 7.0) and DNAs were ethanol-precipitated. The pellets were dissolved in distilled H_2_O and the DNA aliquots (100–300 ng) were used for the PCR amplifications by JumpStart RedTaq DNA polymerase according to manufactur's procedure (Sigma, USA) in 30-μkl reaction mixtures under the following conditions: 94°C, 4 min; 35 amplification cycles (94°C, 30 s; 64°C, 60 s; 72°C, 30 s) and finally at 72°C, 5 min. The *p16INK4a *primers that corresponded to the upper strand of bisulfite-modified DNA and did not contain CpG dinucleotides in the original sequences were used: sense 5' GAG GAG GGG TTG GTT GGT TAT TAG AG (1115) and antisense 5' TAC CTA ATT CCA ATT CCC CTA CAA AC (1418). The positions of the primers are given in base pairs related to the gene sequence [GenBank: X94154 + U12818]. In some cases the combined primer (5' M13+ sense primer) was used for direct sequencing of PCR product [29]. The resulting PCR products were fractionated in low melting agarose (Sigma), purified using QIAEX II kit (Qiagen) and either used for automated sequencing on DNA Sequencer (ABI Prism 3100-Avant Genetic Analyzer) or were cloned into pGEM-T Easy vector (Promega, USA) and then DNAs of individual clone were sequenced. The same total PCR products were used for determination of methylation status of *p16 INK4a *promoter region by of MSP [30]. The primer sequences specific for methylated (sense 5 ' TTA TTA GAG GGT GGG GCG GAT CGC, antisense 5' GAC CCC GAA CCG CGA CCG TAA) and unmethylated (sense 5' TTA TTA GAG GGT GGG GTG GAT TGT, antisense 5' CAA CCC CAA ACC ACA ACC ATA A) variant of *p16INK4a *and the conditions of amplification were as described by Herman et al [30]. The positions of these primers are indicated at the map of *p16INK4 *promoter region (fig. 1a.)

### RT-PCR

The mRNA level of *p16INK4a *gene was determined by reverse transcription combined with polymerase chain reaction (RT-PCR). RT was carried out in 20 μl reaction mixture containing 1 μg of total cellular RNA, treated with DNAase I (Invitrogene), 1× buffer (50 mM Tris, pH 8.3, 61.5 mM KCl, 3 mM MgCl_2_); 1 mM of each of dATP, dTTP, dGTP and dCTP; 20 pmol of random hexamer; 200 U of reverse transcriptase (Superscript II Rnase H^-^, Invitrogen, USA) at 25°C for 10 min and then at 42°C for 50 min. PCR was performed with the primers: sense, 5' AGC CTT CGG CTG ACT GGC TGG; antisense, 5' GCG CTG CCC ATC ATC ATG AC under the following conditions: 94°C for 2 min; 26 cycles (94°C, 30 s; 58°C, 30 s; 72°C, 40 s), and finally at 72°C for 3 min. To control the content of the initial total mRNA, PCRs were carried out with the primers for *GAPDH *(sense 5' ACC ACA GTC CAT GCC ATC AC, antisense 5' TCC ACC ACC CTG TTG CTG TA) and *HPRT1 *(sense 5' CTG GAT TAC ATC AAA GCA CTG, antisense 5' GGA TTA TAC TGC CTG ACC AAG). The conditions of PCR were as follow: 94°C, 3 min; (94°C, 30 s; 58°C, 30 s; 72°C, 1 min,) and 72°C, 10 min, 23 cycles for *GAPDH *and 30 cycles for *HPRT1*. PCR products were resolved on 2% agarose gels in the presence of ethidium bromide and visualized by fluorescence in transmitted ultraviolet light or transferred to membrane (Hybondand-N+, Amersham Biosciences) and probed with *p16INK4a*-specific P^32 ^labeled internal oligonucleotide 5'CTG CCC AAC GCA CCG AAT AGT TAC G [4].

Analysis of the oncogene *E7 *transcripts was performed with the following primers: HPV16 sense, 5' CGG ACA GAG CCC ATT ACA AT; antisense, 5' GAA CAG ATG GGG CAC ACA AT; HPV18 sense, 5' GCT GAA CCA CAA CGT CAC AC; antisense, 5'-GGT CGT CTG CTG AGC TTT CT. The conditions of PCR were as follow: 94°C, 4 min; (94°C, 30 s; 58°C, 30 s; 72°C, 90 s) 35 cycles and 72°C, 6 min.

## Results

### Analysis of the 5' CpG island methylation by MSP and bisulfite-modified DNA sequencing

First the methylation status of the region around the *p16 INK4a *translation start site was analyzed by methylation-specific PCR (MSP) with commonly used primers (see scheme, fig. 1) [30]. Exactly this region of the *p16 INK4a *CpG island was reported to be methylated in many types of tumors including cervical squamous cell carcinomas [3,22-27,30]. The presence of methylated alleles was revealed in 6 out of 26 (23%) squamous cell carcinomas and in 3 out 14 of samples of normal tissues adjacent to tumors. There were methylated alleles in none of the 5 cervical cancer cell lines. Then methylation status of half of tumor and normal samples and 5 cell lines were verified by the bisulfite-based DNA sequencing. The methylation status of 26 CpG dinucleotides localized within the 200 bp-long fragment of DNA around the *p16 INK4a *translation start site was evaluated. Samples containing methylated alleles of gene as determined by MSP are marked by + (fig. 1a). Bisulfite-modified DNA sequencing analysis has not revealed extensive methylation of this region in either 13 carcinomas or 5 carcinoma cell lines. Sequencing analysis of total PCR product generated from this region revealed a varied number of solitary partially methylated CpG sites in DNA of some tumors and carcinoma cell lines, as well as in DNA of some normal tissues. Examples of partially methylated CpG sites are presented on fig. 1b. There was no essential difference in mosaic patterns of partially methylated CpG sites of tumors and normal tissues. In some cases the number of partially methylated CpG sites was greater in normal tissues than in tumors (fig. 1a, samples 2 and 12). Moreover, the number of partially methylated CpG sites in some of MSP-positive carcinomas was less than in MSP-negative carcinomas had (fig 1a, 7T, 10 T and 13T respectively). The distribution of partially methylated CpG dinucleotides within examined region varied in different samples. There was no preferential association of partially methylated CpG dinucleotides with any transcriptional factor binding site. The analysis of the cloned PCR products showed that 5 out of 5 clones contained only unmethylated CpG sites (fig. 1a and 1b, cl. 10T; Caski, cl.; one out of 5 identical clones is presented). These results are compatible with those for the population of DNA molecules (total PCR product) and demonstrate that methylation of certain CpG sites within the *p16 INK4a *5'CpG islands is a rare event. Thus, no extensive methylation of the examined region was revealed in HR-HPV-positive cervical carcinomas, including carcinomas positive by MSP in our hands.

Next we examined methylation status of 3 HpaII recognition sites located around putative multiple sites of p16 transcription initiation (300 bp upstream of translation start site indicated on scheme, fig. 1). It has been demonstrated previously by CAT assay and bisulfite genomic sequencing that methylation of these critical HpaII sites significantly down-regulates p16 promoter activity [4,31]. Analysis of this region by HpaII-methylation-sensitive PCR revealed the presence of methylated alleles only in 1 out of 26 carcinomas. Methylation of these critical HpaII sites was not detected in any cancer cell lines or normal tissues (data are not presented). These findings demonstrate that methylation of critical HpaII sites in the region of putative transcription start sites is a rare event in HPV-positive cervical carcinomas.

### Expression analysis of *p16 INK4a *by RT-PCR

**Figure 2:**
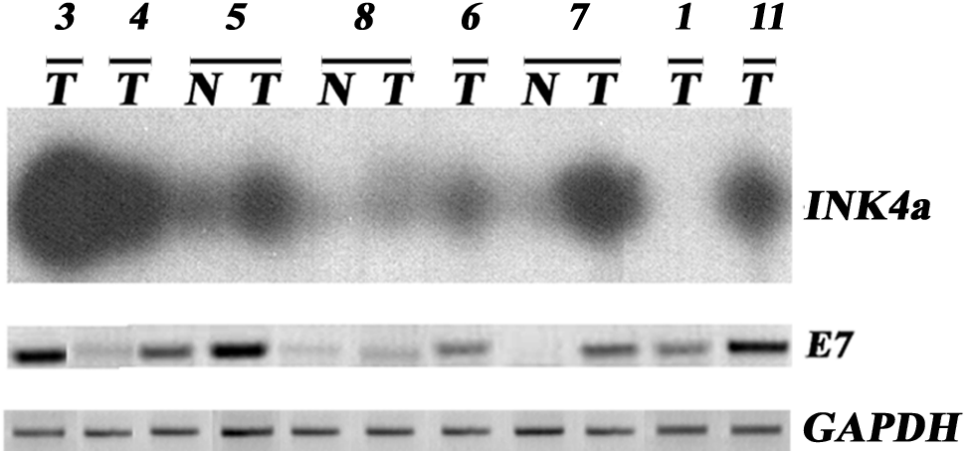
**Expression analysis of *p16 INK4a *in cervical carcinomas by RT-PCR**. Top, electrophoregram after Southern blot hybridization with the *p16INK4a*-specific radioactive probe. Middle and bottom, electrophoregrams after ethidium bromide staining. Middle, RT-PCR with viral *E7 *gene-specific primers as a control for *E7 *expression. Bottom, RT-PCR with the *GAPDH*-specific primers as a control for stability and concentration of RNA. T – carcinomas, N – normal tissue. Specimen numbers above the upper lines are as on fig. 1 and table 1.

To understand whether mosaic methylation of 5'CpG island can modulate *p16 INK4a *transcription, the levels of mRNA were analyzed in all samples listed in fig 1. A representative example of the RT-PCR analysis is shown in fig 2. All carcinomas expressed HPV-E7 oncogene. The up-regulation of *p16 INK4a *transcription was detected in all carcinomas and carcinoma cell lines except for case No 1 (table 1 and fig 2, 1T). Independently of the number of partially methylated CpG sites within 5'CpG island, the mRNA levels were three- to 30-fold higher in carcinomas then in normal adjacent tissues. A rare case of the complete absence of the *p16 INK4a *mRNA in tumor No 1 expressing *E7 *viral oncogene was not related to hypermethylation of the examined regions, which was completely unmethylated in this carcinoma (fig. 1, 1T). Thus, random methylation of some CpG sites in 5'CpG islands does not prevent the *p16 INK4a *transcriptional activation in HR-HPV-positive cervical carcinomas.

### Expression analysis of p16^ink4a ^by immunohistochemistry

**Figure 3:**
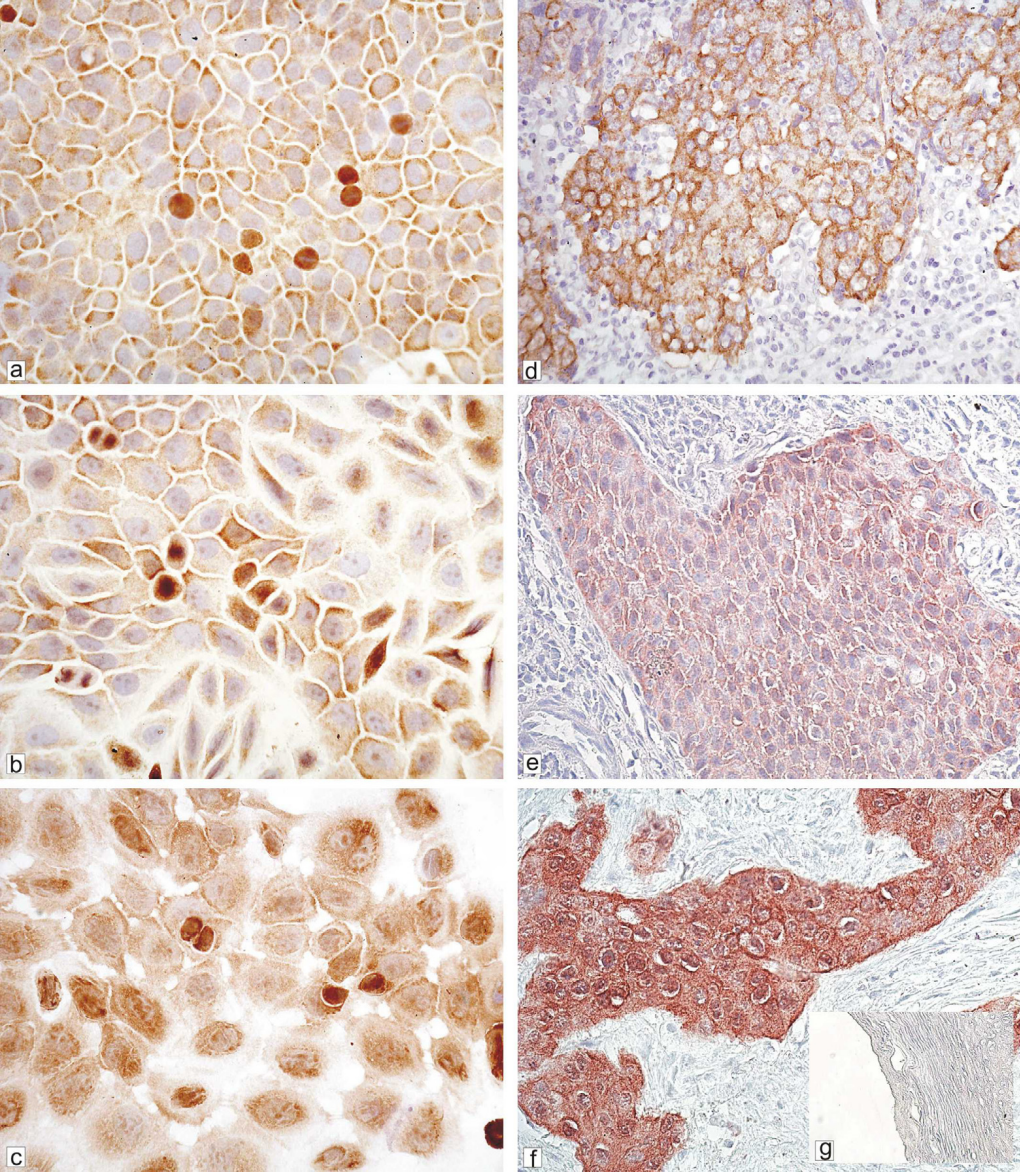
**Immunochemical detection of p16^INK4a ^in cervical carcinoma and cervical cancer cell lines**. Cervical cancer cell lines: **a **– C4-I; **b **– SiHa; **c **– CaSki; primary carcinomas (numbers are indicated as on fig. 1, 2 and table 1): **d **– case 2, **e **– case 6, **f **– case 7, **g **– cervical epithelium adjacent to tumor. Original magnification: a-f × 40; g × 20.

Unlike RT-PCR analysis, the p16^ink4a ^immunostaining allows to estimate the amount of tumor cells expressing the protein. Rpresentative examples of the p16^ink4a ^immunoreactivity are shown in fig. 3 and the results of the analysis are summarized in table 1. Cervical normal cells are not stained for p16^ink4a^, as expected (fig. 3g). On the other hand, the technical procedure used in this study allows to reveal the p16^ink4a ^expression in all HR-HPV-positive cell lines SiHa, HeLa, Caski, C4-I and in HPV-negative line C33A, in which pRb is inactivated by mutation [8]. As expected, a strong predominantly cytoplasmic p16^ink4a ^immunoreactivity was detected in a majority of cells [17]. The relative number of stained nuclei varied in different cell lines from 5 to more than 25% (fig 3a,b,c). A cytoplasmic immunoreactivity in the majority of cells was also revealed for all tumors with *p16 INK4a *transcriptional activation (fig. 3d,e,f) Tumors differed from each other by the number of stained nuclei like cell lines (from less than 1% to more than 25%). Carcinoma No 1 with the complete absence of mRNA did not show immunohistochemically detectable p16^ink4a ^expression either in the cytoplasm or in the nuclei, confirming specificity of the p16^ink4a ^immunoreactivity (table 1 and fig 2, 1T). The tumors that contained methylated alleles of gene (detected by MSP) exhibited the p16^ink4a ^immunoreactivity. In some cases they displayed strong diffuse p16^ink4a ^staining (fig. 3f). The percentage of stained nuclei did not correlate with the mRNA levels. The lowest number of stained nuclei was detected in carcinoma with the highest mRNA level (table 1 and fig. 2, sample 3). Thus, the transcriptional activation of *p16 INK4a *correlates with p16^ink4a ^cytoplasmic staining but does not influence the level of nuclear staining. The random methylation of some CpG sites within 5'CpG islands does not interfere with the p16^ink4a ^immunostaining in the majority of cells of HR-HPV-positive cervical tumors and cancer cell lines.

## Discussion

Bisulfite-modified DNA sequencing analysis demonstrated that there was not extensive methylation within the commonly examined region of the *p16 INK4a *5'CpG island either in13 primary cervical carcinomas or in 5 cancer cell lines. Along with completely unmethylated tumors, some tumors, as well as matched normal tissues, displayed a random location of few partially methylated CpG sites and the lack of completely methylated CpG sites within the examined region. Revealed methylation patterns of the population of DNA molecules and analysis of individual DNA molecules (see fig. 1a, clones) show that hypermethylation of certain CpG sites does not take place in the majority of cells. Our results are consistent with the unique study in which the methylation status of a short DNA fragment within this region has been evaluated by pyrosequencing in cervical intraepithelial neoplasia (CIN). Partial methylation (6–24%) of 1–3 out of 6 CpG sites was demonstrated in MSP-positive CIN [32]. The partial random methylation that has been revealed in some carcinomas and normal tissues cannot exclude the presence of hypermethylated alleles in some cells that can be detected by MSP analysis. MSP is simple, highly sensitive (1 methylated allele in the presence of 10^5 ^unmethylated alleles) and non-quantitative test. A minimal degree of methylation can be registered as "positive methylation" depending on the original DNA concentration, primer sets and the number of cycles used for PCR [32,33]. Indeed in different populationsof patients the *p16 INK4a *methylation was determined in 25–57% of invasive cervical squamous cell carcinomas (I-IV FIGO stages) by MSP [22-27]. Our results of MSP analysis of the same *p16 INK4a *region with the same set of primers in invasive SCC (I-III FIGO stages) are in close agreement with these findings. However methylation patterns determined by bisulfite sequencing show that the *p16 INK4a *5'CpG island hypermethylaion is not typical for the majority of cancer cells in these carcinomas. Thus, methylation cannot be an effective marker of the HR-HPV-positive cancer cells.

It has been demonstrated previously by CAT assay and bisulfite genomic sequencing that methylation of critical CpG sites located around putative multiple sites of the transcription initiation significantly down-regulates the *p16 INK4a *promoter activity. On the contrary, *p16 INK4a *transcription can occur even in the presence of a relatively heavy methylation of CpG sites surrounding the ATG initiation codon (the region commonly examined by MSP) [4,31] Our data are in an agreement with these findings. All cervical tumors and cancer cell lines with partial methylation determined by bisulfite sequencing (including MSP-positive samples) showed high level of *p16 INK4a *mRNA as compared with normal tissues. (All of these carcinomas had no methylation of critical *Hpa*II sites near the transcription start sites). Thus, partial methylation of some CpG sites in the region around the translation start site does not prevent the *p16 INK4a *transcriptional activation. Transcriptional activation of *p16 INK4a *was strongly associated with the HPV-E7 oncogene expression. These findings have confirmed that activation of *p16 INK4a *expression due to the HPV-E7 expression takes place at the level of transcription [7]. We conclude that partial random methylation and the *p16 INK4a *MSP-positivity are compatible with the activation of the transcription due to the HPV-E7 expression. Unfortunately, the *p16 INK4a *transcriptional activation and HR-HPV-E7 oncogene expression were not analyzed in most of the studies demonstrating methylation of *p16 INK4a *by MSP in some portion of cervical carcinomas [22-27]. Only in one of these studies the p16^ink4a ^expression was evaluated by immunohistochemical technique, however the authors did not find any correlation between methylation stutus determined by MSP and immunohistochemical staining of p16^ink4a ^[25].

Transcriptional activation of *p16 INK4a *correlated with p16^ink4a ^cytoplasmic staining in the majority of tumor cells in all samples, but did not correlate with the number of stained nuclei. It is generally believed that p16^ink4a ^functions as Cdk-inhibitor in the nucleus. It has been demonstrated that in normal cells p16^ink4a ^localizes mainly in nuclei [34]. On the contrary, a loss of p16^ink4a ^nuclear staining and retention of cytoplasmic staining have been observed earlier in different tumors including cervical carcinomas and cell lines [17,35,36]. Cytoplasmic staining is controversial and classified as unspecific as well as specific staining in several studies [35-37]. Recently it was demonstrated that p16^ink4a ^was present in protein complexes and bind with Cdk4/6 in both cytoplasmic and nuclear fractions of several cancer cell lines [37]. Authors believe that the p16^ink4a ^cytoplasmic staining is specific. Functional p16^ink4a ^was revealed in cytoplasmic fraction of Rb-functional and Rb-inactivated tumor cells. Mechanism of cytoplasmic p16^ink4a ^accumulation in cancer cells is unknown but it is evident that the accumulation of p16^ink4a ^in cytoplasm of tumor cells occurs independently of a functional status of pRb. Our data are in line with this observation. They demonstrate that cytoplasmic staining and the lack or low number of stained nuclei do not mean the absence of *p16 INK4a *transcriptional activation in HR-HPV-positive cervical carcinomas. In the case of the complete absence of mRNA (carcinoma No 1), the p16^ink4a^-specific antibodies did not reveal any cytoplasmic staining confirming specificity of the p16^ink4a ^immunoreactivity. So, it is impossible to exclude that a strong uniform cytoplasmic staining may indicate the presence of cancer cells with deregulated expression of viral oncogenes, pRb inactivation and *p16 INK4a *transcriptional activation. Specificity of p16^ink4a ^cytoplasmic staining in parallel with transcriptional activation needs to be further investigated. It is possible that further deregulation of the pRb/p16 pathway (lack of p16^ink4a ^transportation to nuclei) appears during progression from CIN to carcinomas. These studies may help to avoid discrepancies in interpretation of the p16^ink4a ^immunoreactivity.

On the other hand, HPV-positive invasive carcinomas in which the HPV oncogene *E7 *is expressed but the p16^ink4a ^overexpression does not occur do exist [[17] and case No 1 in our study]. These carcinomas are rare examples of simultaneous inactivation of both p16^ink4a ^and pRb. Mechanisms of the disruption of the regulatory feedback in pRb/p16 pathway in these carcinomas are not established. The lack of the *p16INK4a *5'CpG island hypermethylation in the carcinoma No 1 suggests that there is another cause of the p16^ink4a^-negativity except hypermethylation.

## Conclusion

The *p16INK4a *5'CpG island hypermethylaion is not a frequent event in HR-HPV-positive cervical carcinomas. Cytoplasmic accumulation of p16^ink4a ^is a feature of cervical carcinomas. Our data confirm other previous studies claiming specific a *p16INK4a *up-regulation in the majority of HR-HPV positive cervical carcinomas at both the protein and mRNA levels. These data demonstrate that the 5'CpG island hypermethylation of the gene can not be an effective marker of HR-HPV-positive cancer cells with up-regulated p16^ink4a ^expression.

## Competing interests

The author(s) declare that they have no competing interests.

## Authors' contributions

TI carried out bisulfite-modified DNA sequencing and MSP analysis. DG and AK carried out the RNA, DNA extraction, HPV-typing and RT-PCR analysis. LZ and GV performed immunohistochemical staining and analysis. YA, LZ and GF were independent pathologist, who contributed to verification of the diagnosis and evaluation of immunohistochemical staining. GF revised the manuscript critically. FL conceived to the study and contributed to study conception and design. NK contributed to study conception and design and prepared the draft of manuscript. All authors read and approved the final version of the manuscript.

## Pre-publication history

The pre-publication history for this paper can be accessed here:



## Figures and Tables

**Table 1:** Methylation and expression of gene *p16 INK4a *in HR-HPV-positive cervical carcinomas and cervical cancer cell lines

Carcinoma/cells line	Methylation status of 5' CpG island **	RT-PCR ***	Immunochemical detection	
			Number of stained nuclei (%)	Cytoplasmic staining
1	1	-	0	*Negative*
2	3	+	<1	
3	4	+		
4	0	+	<5	
5	0	+		*Positive in the majority of cancer cells*
6	4	+	<25	
7	5*	+		
8	7	+		
9	12*	+		
10	8*	+	>25	
11	3	+		
12	0	+		
13	10	+	nt	*nt*

C4-I	0	+	<5	
HeLa	2	+	10–15	*Positive in the majority of cells*
SiHa	6	+		
C33A	0	+		
CaSki	0	+	>25	

* tumors that have methylated alleles, as determined by MSP

** number of partially methylated CpG dinucleotides out of 26 CpG dinucleotides in 5' region of **p16***INK4a *examined by bisulfite-modified DNA sequencing (see fig.1)

*** more than 3 fold increase of the mRNA level in tumor compared to normal tissues
